# Exploration of the relationship between gastric cancer and nutritional risk factors: insights from the Korea National Health Insurance Database

**DOI:** 10.3389/fnut.2025.1538133

**Published:** 2025-05-13

**Authors:** You Na Kim, Chi Young Kim

**Affiliations:** ^1^Department of General Surgery, Ewha Mokdong Hospital, Ewha Womens University School of Medicine, Seoul, Republic of Korea; ^2^Department of Internal Medicine, Yonsei University College of Medicine, Seoul, Republic of Korea

**Keywords:** gastric cancer, nutrients, micronutrients, food, lifestyle

## Abstract

**Background/Objectives:**

Gastric cancer is the third most common cause of cancer-related deaths. Gastric cancer rates vary across regions, which may be attributable to factors such as *Helicobacter pylori* infection, environmental factors, and genetic predispositions. We examined the association between gastric cancer, nutrient intake, and lifestyle parameters in Korean adults.

**Methods:**

This study utilized the KNHANES dataset (2012–2016) to explore the nutritional risk factors associated with gastric cancer. Multivariable analysis was conducted to confirm the association between micronutrients and specific food items using questionnaires designed to collect data on individuals’ consumption frequency and nutrient intake.

**Results:**

This study enrolled 18,894 participants, including 229 diagnosed with gastric cancer. Factors associated with gastric cancer included male sex, older age, low body-mass index, and frequent consumption of food outside the home. Multivariate analysis indicated that a lower intake of protein (odds ratio [OR] 0.98, 95% confidence interval [CI] 0.97–0.99, *p* < 0.001), fat (OR 0.99, 95% CI 0.98–0.99, *p* < 0.004), and thiamine (OR 0.59, 95% CI 0.45–0.76, *p* < 0.001) and a higher intake of niacin (OR 1.05, 95% CI 1.02–1.08, *p* < 0.001) were correlated with an increased risk of gastric cancer. Additionally, specific dietary items, such as tteok (rice cake) and soju, contributed to an elevated gastric cancer risk (OR 1.21, 95% CI, 1.01–1.40; OR 1.14, 95% CI, 1.03–1.25; *p* < 0.001).

**Conclusion:**

We found an association between gastric cancer and various nutritional and lifestyle parameters. Nutrient intake and lifestyle-related factors significantly influence the prevalence of gastric cancer, suggesting that tailored interventions could mitigate this risk in specific populations.

## 1 Introduction

Gastric cancer represents a public health challenge worldwide despite a decrease in its incidence over the last 50 years. According to GLOBOCAN 2023, nearly a million new cases of gastric cancer occur annually, with two-thirds resulting in death (1, 2).

Gastric cancer incidence rates were highest in Eastern Asia for both males and females; specifically, males in Japan (48.1%), Mongolia (47.2%), and Korea (39.7%) exhibited the highest rates globally. Furthermore, Eastern Asia had the highest mortality rates for both males (21.1%) and females (8.8%). Although the incidence of gastric cancer is decreasing in Korea, it remains among the most common malignant tumors worldwide (1, 2).

Previous epidemiological studies have suggested that *Helicobacter pylori* infection and family history play important roles in the etiology of gastric cancer (3–6). Additionally, smoking, occupational exposure, previous gastric surgery, and low socioeconomic status increase the risk of gastric cancer.

Dietary factors linked to a heightened risk of gastric cancer include obesity, alcohol consumption (7), and salt-preserved food consumption (8). In contrast, the consumption of fresh fruits, vegetables, and antioxidant vitamins may decrease the risk of gastric cancer.

Previous studies have shown an elevated risk of gastric cancer associated with high consumption of staple foods, noodles, dumplings, and bread (9, 10). Overeating can be harmful to the stomach and increases the risk of gastric cancer by causing physical damage to the gastric mucosa through repeated stretching of the stomach lining. Moreover, the traditional Korean diet has been linked to gastric cancer. This association could be attributed to the continuous irritation of the stomach wall from the consumption of salty and spicy foods, which potentially contribute to gastric cancer development. Although various other dietary factors have been associated with gastric cancer, the evidence varies and remains inconclusive.

This study aimed to investigate the association between gastric cancer and nutrient intake, socioeconomic factors, and eating habits using data from the Korea National Health and Nutritional Examination Survey (KNHANES).

## 2 Materials and methods

This study was conducted in Korea and was based on the KNHANES dataset (2012–2016), aiming to explore the nutritional risk factors for gastric cancer.

KNHANES is a yearly cross-sectional health and nutritional survey conducted by the Division of Chronic Disease Surveillance of the Korea Centers for Disease Control and Prevention and the Korean Ministry of Health and Welfare. Utilizing a multistage, complex sampling methodology ensures that the survey is representative of the country’s non-institutionalized population. The KNHANES dataset is publicly available on the Korea Centers for Disease Control and Prevention website and is often used for research purposes.

**FIGURE 1:**
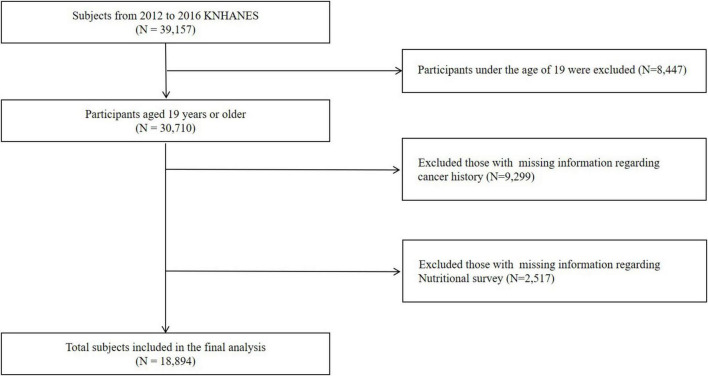
Flow diagram of the study participant selection process.

The initial sample comprised 39,157 participants who completed health interviews, health behavior surveys, and health examination surveys. The exclusion criteria for the study included individuals aged under 19 years (*N* = 8,447), missing values for baseline characteristics and cancer history (*N* = 9,299), and missing nutritional survey data (*N* = 2,517). Ultimately, this study included 18,894 participants (Figure 1).

### 2.1 Nutritional survey data

The current study examined 24-h dietary recall data (KNHANES, 2012–2016) to determine daily food consumption and nutrient intake trends. Through face-to-face interviews, a qualified dietitian visited the participants’ homes to administer the nutrition survey and assess their dietary habits, food consumption frequency, and dietary intake. The survey included information on meal patterns, dining out, family meals, supplement usage, nutrition knowledge, food labeling awareness, and food security. The food frequency questionnaire comprised 63 essential food items that provided energy and nutrients. The food intake questionnaire employed a 24-h recall method, allowing respondents to report their consumption of different dishes and foods, aided by various measuring tools (11).

### 2.2 Statistical analysis

All values are expressed as mean ± standard deviation. Continuous variables were compared using Student’s *t*-test, while categorical variables were analyzed using the chi-square test or Fisher’s exact test, as appropriate. Multivariable logistic regression was performed to estimate odds ratios (ORs) and 95% confidence intervals (CIs), adjusting for potential confounders. Variables included in the multivariable model were selected based on statistical significance in univariate analyses (*p* < 0.05) and clinical relevance supported by prior literature. A *p*-value < 0.05 was considered statistically significant. All statistical analyses were conducted using R software (version 4.3.0; The R Foundation for Statistical Computing, Vienna, Austria).^1^

### 2.3 Ethics approval and consent to participate

This study was approved by the Institutional Review Board of the Yonsei University Gangnam Severance Hospital (Reference No.: 2023-0663-001). The requirement for informed consent from participants was waived because of the anonymity of the data sourced from the KNHANES database. All procedures were conducted in compliance with the approved protocol and adhered to pertinent guidelines and regulations.

## 3 Results

### 3.1 Baseline characteristics

A total of 18,894 individuals were enrolled in our analysis, including patients with gastric cancer and controls. Table 1 presents the participants’ characteristics. The participants’ average age was 51.5 years. Information regarding smoking and alcohol consumption habits was collected through a questionnaire and used as a covariate in the association analyses. The average age of patients diagnosed with gastric cancer was 68.0 ± 9.6, which was notably higher than that of the overall sample. Additionally, the prevalence of gastric cancer was higher among women than among men (62.4 vs. 40.4%, *p* < 0.001). Overall, patients with gastric cancer tended to be placed in the first quartile of household income, and relatively few were placed in the fourth quartile. Similarly, most patients were placed in the lower quartiles of educational attainment, with few placed in the fourth quartile, suggesting lower socioeconomic status. In addition, gastric cancer was significantly more prevalent outside of fasting periods (5.3 ± 1.8 vs. 4.3 ± 1.6, *p* < 0.001). Univariate analysis indicated that age, male sex, body mass index (BMI), alcohol consumption, frequency of eating outside of fasting periods, lower education level, lower household income, and hypertension could be considered risk factors for gastric cancer. Multivariate logistic regression analysis further revealed that the risk factors for gastric cancer included male sex, older age, lower BMI, smoking status, and a high frequency of eating meals outside the home (Table 2).

**TABLE 1:** Baseline characteristics of the study participants.

	Normal	Gastric cancer	Total	*p*-value
	**(*N* = 18,665)**	**(*N* = 229)**	**(*N* = 18,894)**	
Age (years)	51.3 ± 16.8	68.0 ± 9.6	51.5 ± 16.8	< 0.001
Male sex	7,536 (40.4)	143 (62.4)	7,679 (40.6)	< 0.001
BMI, kg/m^2^	23.8 ± 3.5	22.0 ± 2.9	23.8 ± 3.5	< 0.001
**Smoking**				< 0.001
Never	11,609 (62.8)	97 (43.5)	11,706 (62.6)	
Former	391 (2.1)	0 (0.0)	391 (2.1)	
Current	6,480 (35.1)	126 (56.5)	6,606 (35.3)	
**Alcohol**				< 0.001
No	8,882 (48.0)	143 (64.1)	9,025 (48.2)	
Mild	5,865 (31.7)	35 (15.7)	5,900 (31.5)	
Moderate	2,581 (14.0)	20 (9.0)	2,601 (13.9)	
High	1,171 (6.3)	25 (11.2)	1,196 (6.4)	
Hunger time	12.6 ± 2.7	12.7 ± 3.1	12.6 ± 2.7	0.695
**Income**				< 0.001
1st	3,698 (19.9)	94 (41.2)	3,792 (20.1)	
2nd	4,692 (25.2)	53 (23.2)	4,745 (25.2)	
3rd	5,009 (26.9)	48 (21.1)	5,057 (26.9)	
4th	5,199 (28.0)	33 (14.5)	5,232 (27.8)	
**Education**				< 0.001
1st	4,412 (23.8)	118 (52.0)	4,530 (24.1)	
2nd	1,960 (10.6)	27 (11.9)	1,987 (10.6)	
3rd	6,105 (32.9)	51 (22.5)	6,156 (32.8)	
4th	6,078 (32.8)	31 (13.7)	6,109 (32.5)	
Outside fasting	4.3 ± 1.8	5.3 ± 1.6	4.3 ± 1.8	< 0.001
Hypertension	4,519 (24.2)	81 (35.4)	4,600 (24.3)	< 0.001
Diabetes	1,754 (9.4)	31 (13.5)	1,785 (9.4)	0.044

BMI, body mass index.

**TABLE 2:** Association of gastric cancer with baseline characteristics.

	Univariate model		Multivariate model	
	**OR (95% CI)**	***p*-value**	**OR (95% CI)**	***p*-value**
Age	1.08 (1.07–1.09)	< 0.001	1.07 (1.06–1.08)	< 0.001
Female sex	0.41 (0.31–0.53)	< 0.001	0.54 (0.36–0.82)	0.004
BMI, kg/m^2^	0.84 (0.80–0.88)	< 0.001	0.83 (0.79–0.87)	< 0.001
Smoking	1.25 (1.06–1.46)	0.007	1.23 (1.01–1.51)	0.043
Alcohol	0.86 (0.74–1.01)	< 0.001		
Hunger time	1.01 (0.96–1.06)	0.660		
Outside fasting	0.49 (0.42–0.58)	< 0.001	1.22 (1.02–1.47)	0.030
Income	0.63 (0.55–0.71)	< 0.001		
Education	0.57 (0.51–0.64)	< 0.001		
Hypertension	1.71 (1.30–2.24)	< 0.001		
Diabetes	1.51 (1.01–2.18)	0.035		

OR, odds ratio; CI, confidence interval; BMI, body mass index.

### 3.2 Micronutrients

Table 3 presents the intake of vitamins and minerals among the participants who completed the nutritional survey. Notably, individuals with gastric cancer exhibited significantly lower intakes of protein (1,092.5 ± 691.3 vs. 901.1 ± 628.1, *p* < 0.001) and fat (41.8 ± 34.8 vs. 24.8 ± 18.2, *p* < 0.001) compared to controls. In addition, the overall caloric intake (1,981.2 ± 915.6 vs. 1,720.5 ± 628.7, *p* < 0.001) was lower among gastric cancer participants than among the controls.

**TABLE 3:** Comparison of dietary micronutrient intake between participants with gastric cancer and controls.

	Normal (*n* = 18,665)	Gastric cancer (*n* = 229)	Total (*N* = 18,894)	*P*-value
Total energy (Kcal)	1,981.2 ± 915.6	1,720.5 ± 628.7	1,978.1 ± 913.1	< 0.001
Water (g)	1,092.5 ± 691.3	901.1 ± 628.1	1,090.1 ± 690.9	< 0.001
Protein (g)	68.6 ± 42.9	55.1 ± 24.5	68.4 ± 42.8	< 0.001
Fat (g)	41.8 ± 34.8	24.8 ± 18.2	41.6 ± 34.7	< 0.001
Ca (mg)	486.8 ± 320.4	455.3 ± 373.1	486.4 ± 321.1	0.205
P (mg)	1,056.3 ± 554.1	941.9 ± 429.9	1,055.0 ± 552.9	< 0.001
Fe (mg)	16.2 ± 29.8	15.4 ± 10.3	16.2 ± 29.6	0.311
Na (mg)	3,690.2 ± 2,862.1	3,368.0 ± 2,448.9	3,686.3 ± 2,857.6	0.050
K (mg)	2,999.7 ± 1,621.2	2,823.9 ± 1,598.1	2,997.5 ± 1,621.0	0.103
Vit A (μgRE)	698.3 ± 934.3	759.4 ± 1,092.3	699.1 ± 936.3	0.401
Carotene (μg)	3,401.6 ± 4,854.1	3,727.1 ± 5,341.3	3,405.6 ± 4,860.3	0.360
Retinol (μg)	117.5 ± 425.4	124.7 ± 668.7	117.6 ± 429.1	0.871
Thiamine (μg)	1.8 ± 1.0	1.5 ± 0.8	1.8 ± 1.0	< 0.001
Riboflavin (μg)	1.4 ± 0.9	1.1 ± 0.8	1.4 ± 0.9	< 0.001
Niacin (μg)	15.2 ± 10.0	13.2 ± 7.2	15.2 ± 9.9	< 0.001
Vita C (μg)	93.2 ± 112.0	110.7 ± 120.1	93.4 ± 112.1	0.019

Ca, calcium; P, phosphate; Fe, iron; Na, sodium; K, potassium; vit, vitamin.

### 3.3 Nutritional survey for specific food items

We assessed the association between gastric cancer and the weekly dietary frequency of the 30 most consumed food items among participants who completed the nutritional survey (Table 4). Univariate analysis indicated that the weekly dietary frequency of rice, dumplings, rice cake, kimchi stew, stir-fried pork, sundae, chicken, soju, beer, and makgeolli consumption influenced the risk of gastric cancer.

**TABLE 4:** Association of gastric cancer with weekly dietary consumption frequency of the 30 most consumed food items.

Variables	OR (95% CI)	*P*-value	Variables	OR (95% CI)	*P*-value
Rice	0.94 (0.89–0.99)	0.028	Crab sauce	1.15 (0.31–2.00)	0.739
Noodle	1.03 (0.71–1.39)	0.8406	Salted shrimp	1.19 (01.00–1.32)	0.739
Dumpling	0.38 (0.10–0.99)	0.101	Bean sprouts	0.84 (0.61–1.10)	0.267
Bread	0.60 (0.27–1.00)	0.121	Ssam vegetables	1.08 (0.90–1.23)	0.363
Rice cake	1.31 (1.10–1.54)	< 0.001	Garlic	1.08 (0.89–1.24)	0.370
Soybean paste stew	1.10 (0.92–1.25)	0.237	Ssamjang	1.08 (0.93–1.20)	0.253
Kimchi stew	0.50 (0.30–0.75)	0.003	Kimchi	1.01 (0.97–1.05)	0.533
Fried egg	0.90 (0.76–1.05)	0.223	Pickled and fermented vegetables	1.01 (0.88–1.11)	0.885
Pork belly	0.29 (0.13–0.58)	0.001	Roasted seaweed	1.03 (0.93–1.12)	0.494
Boiled pork	0.74 (0.26–1.47)	0.511	Milk	0.91 (0.80–1.01)	0.099
Stir-fried pork	0.40 (0.16–0.81)	0.028	Coffee	0.98 (0.95–1.01)	0.217
Beef	0.68 (0.24–1.38)	0.386	Soft drink	1.06 (0.87–1.22)	0.499
Sundae	0.04 (0.00–0.34)	0.010	Soju	1.21 (1.10–1.30)	< 0.001
Chicken	0.03 (0.00–0.17)	< 0.001	Beer	0.56 (0.31–0.84)	0.020
Salad	1.06 (0.87–1.22)	0.499	Makgeolli	1.21 (1.00–1.36)	0.007
Mackerel	1.16 (0.80–1.52)	0.371	Fruits (average)	1.07 (0.66–1.87)	0.800

OR, odds ratio; CI, confidence interval.

### 3.4 Association of gastric cancer with micronutrients and food items for multivariate analysis

In multivariate logistic regression analysis, the risk of gastric cancer remained higher in the group with low intake of protein (OR 0.98, 95% CI 0.97–0.99, *p* < 0.001) and fat (OR 0.99, 95% CI 0.98–0.99, *p* < 0.004) (Table 5). For micronutrients, we observed an association between lower intake of thiamine (OR 0.59, 95% CI 0.45–0.76, *p* < 0.001) and higher intake of niacin (OR 1.05, 95% CI 1.02–1.08, *p* < 0.001) and gastric cancer risk. However, sodium and potassium intake did not appear to affect the incidence of gastric cancer.

**TABLE 5:** Association of gastric cancer with micronutrients and food items.

Micronutrients	OR (95% CI)	*P*-value	Food	OR (95% CI)	*P*-value
Protein (g)	0.98 (0.97–0.99)	< 0.001	Rice cake	1.21 (1.01–1.40)	0.012
Fat (g)	0.99 (0.98–0.99)	0.004	Soju	1.14 (1.03–1.25)	0.009
Thiamine (μg)	0.59 (0.46–0.77)	< 0.001	Dumpling	1.88 (0.77–3.25)	0.069
Niacin (μg)	1.05 (1.02–1.08)	< 0.001			

Multivariate logistic regression adjusted for age, sex, smoking, outside fasting. OR, odds ratio; CI, confidence interval.

In the case of specific food items, multivariate analysis revealed a significant association between the consumption of rice cake and soju and the risk of gastric cancer (OR 1.21, 95% CI 1.01–1.40; and OR 1.14, 95% CI 1.03–1.25; *p* < 0.001).

## 4 Discussion

In this study, we identified the associations between gastric cancer and various nutritional and lifestyle factors in a nationally representative sample of Korean adults. Our findings underscore the role of factors such as advanced age, male sex, BMI, socioeconomic status, and eating outside fasting periods in the association with gastric cancer. Moreover, reduced protein and fat intake, thiamine deficiency, and increased consumption of rice cake and soju have been identified as factors associated with gastric cancer risk.

Previous studies have suggested that the incidence of gastric cancer is associated with environmental factors, including socioeconomic status, occupational exposure, alcohol consumption, smoking, dietary habits, and consumption of salt-preserved foods (12–15). Among these factors, dietary intake showed the strongest association with gastric cancer. In previous studies, a high-salt diet has been linked to the risk of gastric cancer (16), whereas a high consumption of fresh fruits and vegetables has shown protective effects (17), consistent with the findings of our study. However, we found no association between sodium or potassium intake and the risk of gastric cancer.

A previous meta-analysis reported a 17% increase in gastric cancer risk when salted fish were consumed and a 15% increased risk per 40 g of pickled vegetables consumed (18). Bouras et al. (19) suggested that a high intake of salted fish was associated with an increased risk of gastric cancer, whereas the consumption of salt itself and that of several other high-salt products showed nominal associations. This study revealed no effect of either sodium or potassium intake on gastric cancer risk; however, multivariate analysis results showed that soy and kimchi stew intake were associated with the disease. However, accurately measuring the total amount of salt consumed in an individual’s diet is difficult and frequently subject to error when conducted within a standard epidemiological study setting (20). The higher consumption of sodium by Koreans compared to other groups may be attributed to soup-based diets (21, 22). The mechanisms responsible for the observed associations are complex and involve more than just sodium chloride. High consumption of salt-preserved foods may be an indicator of poor diet quality and low socioeconomic status, which might increase susceptibility to *H. pylori* infection and hence increase the risk of gastric cancer (23).

Smoking and alcohol consumption are recognized as carcinogenic factors (24–26). Smokers or alcohol drinkers prefer salty food (22), whereas smokers have a low intake of fruit and dairy products and a high intake of fast food (27). Previous studies have shown a dose–response relationship between the number of cigarettes smoked and the risk of gastric cancer (28, 29). Moreover, former male (but not female) smokers tend to have an increased risk of gastric cancer. Furthermore, the shorter the interval since quitting smoking, the higher the risk of gastric cancer, even after accounting for cigarette smoking years. In our study, we observed an association between smoking and gastric cancer. The mechanisms underlying this relationship remain unclear, although several mechanisms have been proposed. Nitrosamines and other nitroso compounds found in cigarette smoke may play a role in the development of gastric cancer (30, 31). Cigarette smoking has also been associated with an increased risk of transitioning to dysplasia and intestinal metaplasia, which are key steps in gastric carcinogenesis (32). In addition, a randomized crossover study demonstrated that smoking could delay stomach emptying and gastroesophageal reflux disease, which are closely associated with the development of cardiac cancer (33, 34).

The impact of alcohol on gastric cancer involves the generation of inflammatory markers and oxidative stress compounds induced by inflammation, such as oxygen radical species (24). Alternatively, ethanol, being soluble in fat, could have potentially detrimental effects on the gastric mucosa (35). A previous study reported that the most popular type of alcoholic beverage in Korea is soju (58.2%), followed by beer (35.6%) (36). Kim et al. found that individuals who consumed soju exhibited a heightened risk of gastric cancer, although the association did not reach statistical significance (36). Contrary to previous findings, we have shown a statistically significant correlation between both beer and soju consumption and gastric cancer risk in Korean patients.

Our results also suggest an association between tteok (rice cake) consumption and gastric cancer. Traditional Korean rice cakes, known as “tteok,” are generally perceived as healthy foods because they are made from rice and often include various nutritious ingredients such as nuts, beans, and pumpkin. They are typically steamed until the texture becomes light and chewy. However, the health benefits of rice cakes remain controversial. While predominantly composed of carbohydrates and not previously reported to be directly associated with gastric cancer, rice cakes have been linked to gastrointestinal conditions such as peptic ulcer disease and intestinal obstruction, which may be attributed to their glutinous texture and tendency to delay gastric emptying (37, 38). Although rice cakes are widely regarded as a healthy food, their glutinous nature and potential to delay gastric emptying may contribute to mucosal irritation. This repeated irritation could promote gastric carcinogenesis, particularly in individuals with underlying risk factors. Given that rice is the primary ingredient, our findings align with previous reports that frequent meals or dietary patterns centered around rice may be associated with gastric cancer (39). Nevertheless, due to the cross-sectional design of this study, we cannot infer a causal relationship, and further longitudinal studies are warranted to clarify these associations and underlying mechanisms.

This is the first study to compare the consumption patterns of micronutrients and dietary components, including food types, between individuals with gastric cancer and healthy controls. Our results specifically emphasize that primary dish groups are potential contributors to gastric cancer in the Korean population, suggesting a previously unreported association between disease onset and the consumption of soju and tteok. Furthermore, we found that the risk of gastric cancer was correlated with factors such as older age, male sex, lower BMI, smoking, and frequent food consumption outside the home.

Our study had several limitations. First, we were unable to account for the *H. pylori* status of the study participants. Second, the study design was cross-sectional rather than longitudinal, limiting our ability to discuss the associations between risk factors and exposure over time. Third, the number of participants with gastric cancer included in the study was lower than expected based on the national prevalence estimates. This could be attributed to the characteristics of the KNHANES dataset, which primarily consists of individuals undergoing regular health screenings and may have a bias toward those with better health outcomes. Volunteers for research studies and individuals undergoing routine health checkups often have a higher socioeconomic status, potentially introducing selection bias and influencing the study findings.

In summary, we found an association between gastric cancer, various nutrients, and lifestyle parameters in a nationally representative sample of Korean adults. This study highlights the importance of considering individual factors, such as advanced age, male sex, low body mass index, and lower intake of protein and fat, alongside higher intake of niacin and increased consumption frequency of soju and tteok, in assessing the risk of gastric cancer. Nutrient intake and lifestyle-related factors play pivotal roles in the prevalence of gastric cancer, suggesting that tailored interventions could potentially mitigate gastric cancer risk in specific populations.

## Data Availability

The raw data supporting the conclusions of this article will be made available by the authors, without undue reservation.

## Footnotes

1
https://www.r-project.org/
